# Some Partial Impressions

**Published:** 1905-10

**Authors:** S. E. Davenport

**Affiliations:** New York


### SOME PARTIAL IMPRESSIONS.[1]

BY DR. S. E. DAVENPORT, NEW YORK.

THE advantages possessed by modelling composition over plaster, in some cases, are well understood and appreciated by most practitioners, and, in spite of a rather general feeling to the contrary, the successful handling of composition requires quite as much skill as does plaster.

Among the disadvantages of plaster as an impression material, mention may be made of its lack of firmness and consequent failure to press the soft parts into the position they would naturally occupy under a plate.

The greater one's experience with modelling composition, the more positive is the opinion, I think, that the best results are to be obtained when the least thickness possible of the softened material is used.

This means one of two things, either the cup must conform very closely to the mouth or a preliminary impression of composition must be taken and, after being chilled out of the mouth, cut away slightly over the whole surface for the addition of a thin layer of soft composition for the final accurate impression, the

[1] Read before The New York Institute of Stomatology, June 6, 1905.

hard composition underneath serving merely as an addition to the cup to force the soft composition into all inequalities.

While plaster is a more reliable material for impressions of hard upper jaws, when the plate decided upon is to depend for its retention upon atmospheric pressure, the very soft upper gums occasionally met with may be more accurately duplicated by skilful use of modelling composition, and the resultant "suction" will therefore be greater.

It has been my custom for a number of years to use composition almost entirely for the taking of difficult impressions for lower partial plates, the method being the same whether the plate was to be of gold, rubber, or other material.

A composition impression is first taken in a cup which conforms as closely to the desired parts as will any ready-made cup. This composition impression is then poured with plaster as usual, and the resultant model is used for the making of a special cup which will quite accurately fit the parts to be duplicated.

These special impression cups may be made in several ways, two of which I have found most useful.

Exhibit No. 1 represents a swaged cup. A die and counter-die having been made from the plaster model of the preliminary impression, copper plate No. 28 is swaged, then thickened and stiffened by the addition of pure tin flowed over the reverse side with a soldering iron. A wire handle is soldered on, as represented in the one exhibited.

Exhibit No. 2 shows a cast impression cup of pure tin. Upon the plaster model from the preliminary impression a wax form is made of the size and thickness desired, the wire handle being bent and placed in its proper position in the wax. This form, being removed from the plaster model, is then invested with plaster and sand in an ordinary vulcanizing flask in two parts, the wax form in the lower part, the wire handle in the upper.

The flask then being separated and the wax removed, two holes are made through the plaster and sand in the upper half of the flask, connecting from the top of the plaster and sand, the metal cover of the flask being left off, with each extremity of the cavity previously occupied by the wax form.

The melted tin then being poured into one of the holes until it oozes from the other, the cup is made, needing only some trimming and smoothing.

Before taking the final impression, the new impression cup, which already fits the first model, should be carefully adjusted to the mouth so that it will easily go to place by the use of a certain practical movement, and if the design is carried out, the cup will fit the gum and necks of the teeth so accurately that but a thin layer of composition will be necessary.

In taking the impression the cup and composition should be carried to place with considerable force, which should be as evenly distributed over the whole surface as is possible.

No effort should be made at this time to secure an accurate impression of the crowns of the teeth, it being unnecessary to have them represented at this stage of the process, whether the plate is to be of gold or rubber.

If gold is to be used, the plaster teeth would be cut from the model before it is moulded in the sand; and if the plate is to be of rubber, the better way in these difficult cases is to vulcanize the plate upon the model first, using it then for taking the bite, as we would a wax form or a gold plate.

I am reminded of a recent case in which I was asked to make a complicated lower partial plate to take the place of a poorly fitting rubber plate, which the patient had vainly attempted for two or three years to get good use from.

As I was about to take the preliminary impression to have the special cup made the thought came to me that I might use the old plate for the cup. Then adding a thin layer of composition to its under surface, I carried it to the proper position, and the plate made upon that model fit like the proverbial "duck's foot in the mud."

Emphasis should again be given to what in my use of composition has been an established fact: the greatest accuracy of result is obtained with the thinnest possible quantity of the softened material carried to its place in a positive and forceful manner.

